# Ectopic Expression of a Salt-Inducible Gene, *LcSAIN3*, from Sheepgrass Improves Seed Germination and Seedling Growth under Salt Stress in Arabidopsis

**DOI:** 10.3390/genes12121994

**Published:** 2021-12-16

**Authors:** Xiaoxia Li, Weiguang Yang, Junting Jia, Pincang Zhao, Dongmei Qi, Shuangyan Chen, Li Cheng, Liqin Cheng, Gongshe Liu

**Affiliations:** 1 Key Laboratory of Plant Resources, Institute of Botany, Chinese Academy of Sciences, Beijing 100093, China; lixx2013@ibcas.ac.cn (X.L.); qidm@ibcas.ac.cn (D.Q.); sychen@ibcas.ac.cn (S.C.); liugs@ibcas.ac.cn (G.L.); 2 College of Animal Science and Veterinary Medicine, Helongjiang Bayi Agricultural University, Daqing 163000, China; anda580@163.com; 3 Guangdong Provincial Key Laboratory for Crop Germplasm Resources Preservation and Utilization, Agro-Biological Gene Research Center, Guangdong Academy of Agricultural Sciences, Guangzhou 510640, China; jiajunting123@126.com; 4 College of Management Science and Engineering, Hebei University of Economics and Business, Shijiazhuang 050062, China; zhaopincang@163.com; 5 Institute of Hulun Buir Forestry and Grassland Science Research, Hunlunbeier 021008, China; cheng1010li@sina.com

**Keywords:** sheepgrass, chloroplast, salt stress, seed germination

## Abstract

Sheepgrass is a perennial native grass species in China, and it can tolerate high levels of salt stress with an aggressive and vigorous rhizome system. Many salt-stress-responsive genes have been identified in sheepgrass. In this study, we report the cloning and characterization of a novel salt-induced gene, *LcSAIN3* (*Leymus chinensis* salt-induced 3), from sheepgrass. Expression analysis confirmed that *LcSAIN3* was induced by PEG, ABA, and salt treatments, and the expression of *LcSAIN3* was significantly increased in salt-tolerant germplasms under salt treatment. Subcellular localization analysis indicated that the GFP-LcSAIN3 protein was mainly localized in the chloroplasts. The heterologous expression of *LcSAIN3* in Arabidopsis increased the seed germination rate of transgenic plants under salt, ABA, and mannitol treatments. The seedling survival rate, plant height, and fresh weight of the transgenic plants were higher than those of WT plants under salt stress. The overexpression of *LcSAIN3* caused a relatively high accumulation of free proline, enhanced SOD activity, and led to the upregulation of several stress-responsive genes such as *AtRD26*, *AtRD29B*, *AtSOS1,* and *AtP5CS1*. These results suggest that *LcSAIN3* could be a potential target for molecular breeding to improve plants’ salt tolerance.

## 1. Introduction

Soil salinization is a severe problem that affects plant growth, development, and productivity worldwide [1]. The effects of salt on plants lead to osmotic, ion toxicity, and oxidative stress [1,2,3]. Changes in physiological, biochemical, cellular, and molecular processes in plants under salt stress have been investigated by many researchers [2,4,5,6,7], while genetic sources for salt tolerance development in crops have also been studied [8]. Extensive numbers of transcription-factor-encoding genes have been identified in response to salt stress, including DREB, bZIP, NAC, and MYB family genes [9,10,11,12,13], and overexpressing these genes can enhance salt stress tolerance in transgenic plants [2,14,15,16,17]. Previous studies have suggested that the transcription factors can activate many stress-induced genes, such as LEA genes (*RD26*, *RD29A*, *RD29B*, and *RAB18*) and proline biosynthesis genes (*P5CS*), and that LEA proteins are mainly involved in protection to desiccation by acting as cellular dewatering protectants under stress conditions. Proline plays an important role in osmoregulation and can also be used as an active oxygen scavenger to stabilize protein and membrane structures under pressure [2,18,19]. Molecular regulatory networks related to salt stress are complex and have not been fully explored [20]; thus, mining key and novel salt-tolerance-related genes is required for developing breeding strategies to enhance salt stress tolerance in crops.

Sheepgrass (*Leymus chinensis* (Trin.) Tzvel) is a perennial gramineous plant species belonging to the *Leymus*, Triticeae, and Poaceae classification groups and is widely distributed on the eastern Eurasian steppe [21]. This species can survive when the soil moisture content is less than 6% in the dry season and grows well in environments of 600 mmol/L NaCl and 175 mmol/L Na_2_CO_3_ [22,23,24]. Many stress-induced genes from sheepgrass have been identified and characterized using transcriptome sequencing, including *LcDREB2*, *LcDREB3a*, *LcDREB21*, *LcMYB1*, *LcWRKY5*, *LcP5CS*s, *LcbHLH92,* and *LcSAMDC*s [16,25,26,27]. In addition, several novel genes were discovered in sheepgrass, such as the *LcSAIN1* and *LcSAIN2* genes, which can improve the greening rate of cotyledons, root elongation, plant height, and survival rates under salt stress in their transgenic plants [28,29]; the ectopic expression of *LcFIN1* and *LcFIN2* significantly increases freezing stress tolerance in transgenic Arabidopsis and rice [24,30].

Here, we characterize a novel gene, *LcSAIN3,* from sheepgrass; *LcSAIN3* has low homology with *LcSAIN1* and *LcSAIN2*. The expression of *LcSAIN3* is induced by salt stress and can improve the salt tolerance of transgenic Arabidopsis. Thus, we propose that *LcSAIN3* plays an important role in the salinity stress regulatory network.

## 2. Materials and Methods

### 2.1. Plant Materials, Growth Conditions, and Stress Treatment

Sheepgrass variety Zhongke No. 1 and sheepgrass germplasms with different salt tolerances (salt-tolerant germplasm G53, G25, and G16 and salt-sensitive germplasm G13 and G87) were cultivated by the Institute of Botany, the Chinese Academy of Sciences. Sheepgrass, *Arabidopsis thaliana* (ecotype Columbia (Col-0)), and tobacco (*Nicotiana benthamiana*) were grown in a soil mixture of peat moss and vermiculite (2:1, *v*/*v*) in a glasshouse, and the plants’ growth conditions were followed as previously described [23,28,29]. For the analysis of specific expression in different tissues, the roots, stems, leaves, and seeds were collected from two-year-old sheepgrass plants under normal conditions. For salt, ABA, and dehydration treatments, 400 mM NaCl, 100 mM abscisic acid (ABA), and 20% PEG6000 were applied to 4-week-old sheepgrass seedlings as previously described [23,28,29]. A total of 40 plants for each sample were harvested at 0, 1, 3, 5, 12, and 24 h after treatments, and three independent sets of samples were collected for each time point. Samples were immediately frozen in liquid nitrogen and stored at −80 °C.

### 2.2. Cloning and Sequence Analysis of the LcSAIN3 Gene

*LcSAIN3* gene (GenBank ID: MN901606) was isolated from sheepgrass and encoded an unknown functional gene. Total RNA was extracted from 4-week-old sheepgrass seedlings under 400 mM NaCl for 12 h using a TRIzol kit (TaKaRa, Dalian, China) based on the protocol instructions of the manufacturer, and first-strand cDNA synthesis was performed with a SMART RACE cDNA Amplification Kit (Clontech, Palo Alto, CA, USA) according to the manufacturers’ instructions. Full-length *LcSAIN3* cDNA was amplified using the primers 5′-GTAGCCCGTGAGGAAGTT-3′ and 5′-CACTAGAAGGGCCCCGAA-3′, with the cDNA of 5′ RACE used as a template. The amplification was carried out following a previously described method [23,28,29], and all of the PCR products were cloned into a pMD19-T vector and sequenced at Sangon Biotech (Shanghai Co., Ltd., Shanghai, China). 

The *LcSAIN3* sequence was analyzed using the BLAST program of the National Center for Biotechnology Information (NCBI) (https://www.ncbi.nlm.nih.gov/, accessed on 3 January 2020), and subcellular localization was predicted using the Plant-mPLoc program [31].

### 2.3. qRT-PCR Analysis

Total RNA from Arabidopsis and sheepgrass seedlings was isolated using a TRIzol kit (TaKaRa, Dalian, China) according to the instructions of the manufacturer. Total RNA was reverse-transcribed using a PrimeScript^TM^ PCR Kit, and the SYBR^®^ PrimeScript TM PCR Kit was used for qPCR following the manufacturer’s instructions (TaKaRa, Dalian, China). qPCR was performed using the Roche Light Cycler 480 II (Basel, Switzerland), and the qPCR program was set according to our previously described reports [23,28,29]; the data were quantified using the comparative 2^−^^ΔΔ^^CT^ method, as described previously [32]. *LcActin* and *AtActin2* were used as internal controls for assessing the expression levels in sheepgrass and Arabidopsis, respectively. The primers of *LcSAIN3* for qRT-PCR were 5′-ACTGGTGTTGGATGATGAGCG-3′ and 5′-CGGGAGGAAAGATAGAGGTCG-3′. The primers’ information for salt-induced genes in Arabidopsis was followed, as previously described [28,29].

### 2.4. Subcellular Localization of LcSAIN3

The open reading frame (ORF) of *LcSAIN3* was inserted into the expression vector pMDC45 (containing GFP) using Gateway cloning while the construct was transferred to *Agrobacterium tumefaciens* EHA105. The *Agrobacterium tumefaciens* containing the pMDC45-LcSAIN3 (35S::GFP-LcSAIN3) recombinant vector and the pMDC45 empty vector were injected into 4-week-old wild-type tobacco (*N. benthamiana*) leaves, as described by Gao et al. [24]. GFP fluorescence in tobacco leaves was observed and imaged for 2 to 3 d after infiltration using an argon laser at 488 nm (GFP) and 382 bright-field images by confocal microscope Leica TCS SP5 microscope (Leica Microsystems, Wetzlar, Germany) [30]. 

### 2.5. Construct Creation and Plant Genetic Transformation

The ORF of *LcSAIN3* was cloned into a pSN1301 vector, and the construct pSN1301-*LcSAIN3* was introduced into the *Agrobacterium* EHA105. Then, the *Agrobacterium* EHA105 containing the recombinant plasmid was transformed into Arabidopsis following a previously described method [33]. The candidate transgenic Arabidopsis seeds were firstly screened on Murashige and Skoog (MS) solid medium agar supplemented with 30 μg mL^−1^ hygromycin and then further confirmed by PCR analysis using the gene-specific primers.

### 2.6. Phenotypic Analysis of Transgenic Plants

The T3 generation *LcSAIN3* transgenic Arabidopsis lines (line 5, line 6, and line 8) were used for further analysis. The seeds were incubated at 4 °C for 2 d to break dormancy and then germinated on MS medium supplemented with different concentrations of ABA (1 and 2 μM), mannitol (200 and 300 mM), and NaCl (100, 125, 150, 175 and 200 mM), respectively. The germination rate was calculated daily for 7 d by observing radical protrusion, and at least 120 seeds from each transgenic line were evaluated. To test the salt tolerance of transgenic Arabidopsis, 3-week-old plants were treated with 200 mM NaCl for 3 weeks at 3-day intervals, as described previously [34]. Three-week-old transgenic and WT seedlings grown in the MS liquid medium were treated with 150 mM NaCl after 1 d and were sampled and used for qPCR analysis. Additionally, *AtActin* was used as a reference gene.

### 2.7. Measurement of Proline Content and (Superoxide Dismutase) SOD Activity

Proline was measured as previously described [35], and total superoxide dismutase (SOD) activity was measured using nitro blue tetrazolium (NBT) reduction, as described previously [30,36].

### 2.8. Statistical Analysis

The data concerning Arabidopsis seed germination rates and seedling growth parameters, proline content, and SOD activity were subjected to one-way ANOVA using the SPSS 21.0 program (IBM, Chicago, IL, USA).

## 3. Results

### 3.1. Isolation and Sequence Analysis of LcSAIN3 

**Figure 1:**
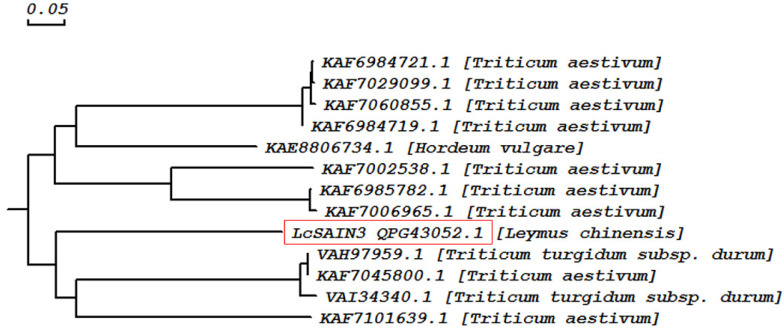
Phylogenetic analysis of LcSAIN3 and homologs was constructed based on amino acid sequences using the neighbor–joining method.

Our previous studies identified many stress-induced genes from sheepgrass using transcriptome sequencing techniques [23]. In this study, the full-length cDNA was obtained from sheepgrass by RACE. The gene (GenBank ID: MN901606) is 847 bp long and encodes 198 amino acids, and BLAST analysis shows it has high homology (72%) with a wheat cDNA clone, WT004_K04 (GenBank ID: AK331493). The amino acid sequence shows 52% homology with the predicted protein product of *Triticum turgidum* subsp. *Durum* (GenBank ID: VAH97959.1) and 40.52% with hypothetical protein CFC21_054876 of *Triticum aestivum* (GenBank ID: KAF7060855.1) using a BLASTX search (Figure 1). Furthermore, it shared low homology to LcSAIN1 (9.67%) and LcSAIN2 (21.93%) (Figure S1). These results imply that it is a novel protein with unknown function, and it was named *LcSAIN3* (*L. chinensis* salt-induced 3).

### 3.2. Expression Analysis of LcSAIN3

**Figure 2:**
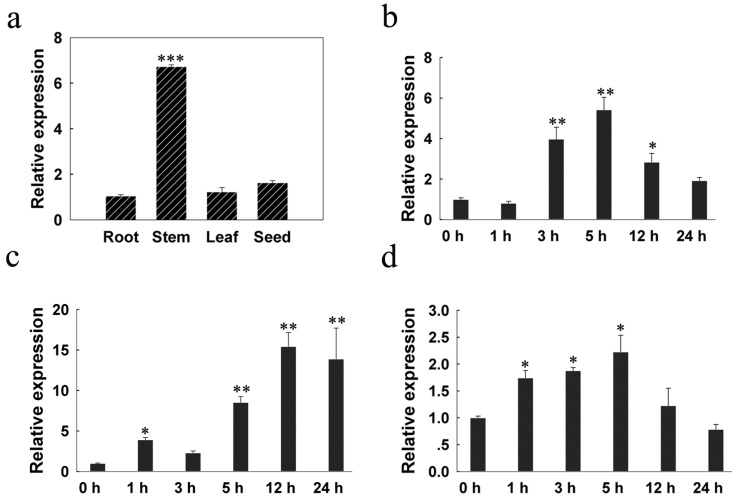
Expression patterns of *LcSAIN3* in sheepgrass tissues and abiotic stress. (**a**) Expression of *LcSAIN3* in roots, stems, leaves, and seeds. (**b**–**d**) Expression patterns of the *LcSAIN3* gene under salt, ABA, and PEG treatments. The sheepgrass *Actin* gene is used as the internal reference gene for normalization, and data represent means ± SDs of three independent biological replicates. ***, **, and * indicate significant differences at *p* < 0.001, *p* < 0.01, and *p* < 0.05, respectively.

The qRT-PCR analysis showed that *LcSAIN3* was highly expressed in the stems of two-year-old sheepgrass plants under normal conditions (Figure 2a). To determine whether *LcSAIN3* was responsive to stress in sheepgrass, 4-week-old sheepgrass seedlings were exposed to salt, ABA, and dehydration stresses and were sampled at different time intervals. QRT-PCR results showed that the expression level of *LcSAIN3* was significantly upregulated at 3 h and reached the highest level after 5 h of NaCl treatment, approximately 5.4-fold (Figure 2b). It is different from the quick response of salt stress treatments; the expression of *LcSAIN3* slowly increased up to 15.5-fold at 12 h and 2.2-fold at 5 h of treatment with ABA and PEG, respectively (Figure 2c,d).

### 3.3. Subcellular Localization of LcSAIN3

**Figure 3:**
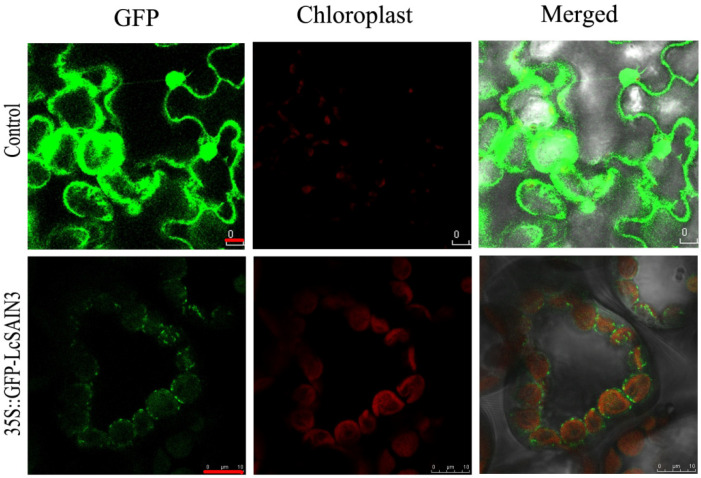
The subcellular localization of LcSAIN3. The GFP signals arising from the GFP control (upper) and 35S::GFP-LcSAIN3 fusion protein (bottom lane) expressed in tobacco leave cells were detected under fluorescent-field illumination, chloroplast autofluorescence, and an overlay using a confocal microscope. Red signals represent chloroplast autofluorescence. Scale bar = 10 μm.

To determine the subcellular localization of LcSAIN3, the protein was predicted by the Plant-mPLoc program (http://www.csbio.sjtu.edu.cn/bioinf/plant-multi/, accessed on 3 January 2020); it demonstrated that LcSAIN3 was a chloroplast protein. To determine the actual subcellular localization of LcSAIN3 in vivo, the ORF sequence was inserted into a pMDC45 vector fused to a GFP reporter gene under the control of the CaMV 35S promoter, and the construct of 35S::GFP-LcSAIN3 was infiltrated into tobacco (*Nicotiana tabacum*) leave cells (using 35S-GFP as the control). As shown in Figure 3, the green fluorescent signals from the 35S::GFP-LcSAIN3 fusion protein and autofluorescent signals of chloroplasts were merged together; these results demonstrate that LcSAIN3 is a chloroplast-localized protein.

### 3.4. Overexpression of LcSAIN3 in Arabidopsis Improves Seed Germination under Salt Stress

**Figure 4:**
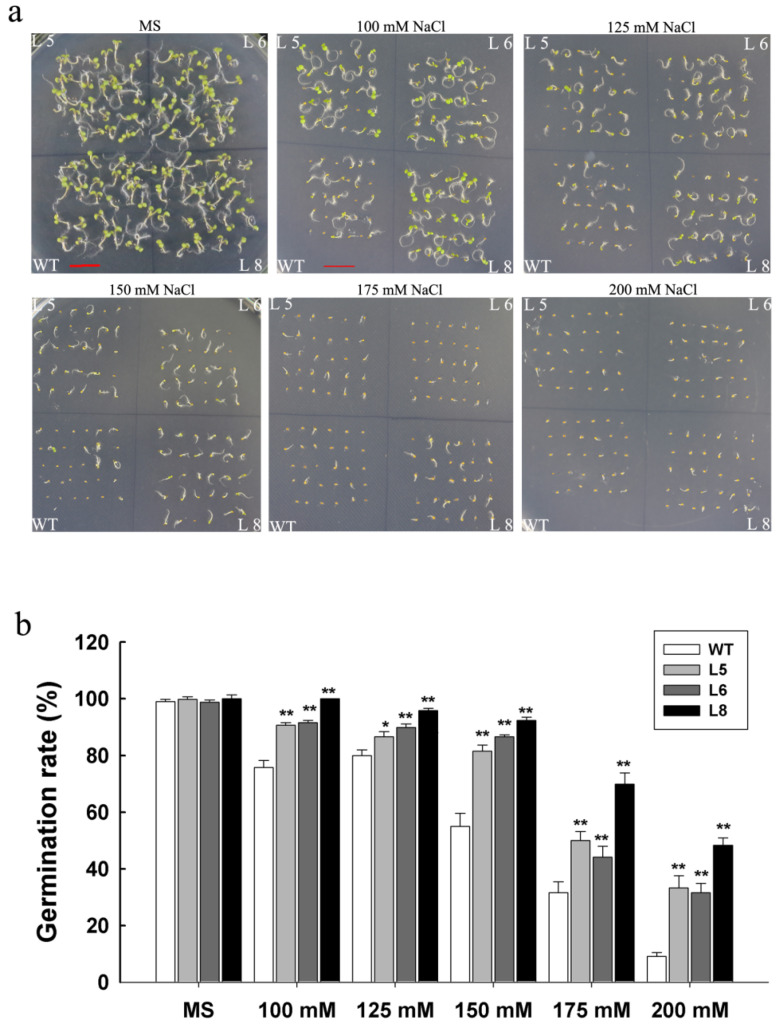
Salt stress tolerance of *LcSAIN3* transgenic Arabidopsis plants at germination stage. (**a**) Phenotypes of WT (wild-type) and *LcSAIN3-*overexpressing lines (L5, L6, and L8 seeds) in MS medium with 0, 100, 125, 150 175, or 200 mM NaCl. (**b**) Seed germination rate under salt stress. Thirty seeds were allowed to grow for seven days after sowing, and each column represents an average of three replicates, and the bars indicate standard deviations (SDs). ** and * indicates significant differences in comparison with the control at *p* < 0.01 and *p* < 0.05. Scale bar = 1 cm.

To further understand the function of *LcSAIN3* in response to salt stress, the Arabidopsis *LcSAIN3*-overexpressed lines were generated. Three T3 transgenic lines (L5, L6, and L8) with higher *LcSAIN3* transcript levels by qRT-PCR were selected for further investigation (Figure S2). To evaluate the performance of *LcSAIN3* transgenic plants in response to salt stress, the seeds of WT and *LcSAIN3-*overexpressed plants were germinated on MS media supplemented with different concentrations of NaCl (0, 100, 125, 150, 175, and 200 mM) after 2 d of stratification. As shown in Figure 4, no obvious differences were detected between WT and transgenic plants on MS medium without NaCl. However, the germination rates of the *LcSAIN3*-overexpressing lines were significantly higher than those of WT plants in the presence of NaCl (Figure 4a). In addition, three transgenic lines (L5, L6, and L8) showed significantly higher germination rates (92%, 86%, and 81%, respectively) than WT plants under 150 mM NaCl (~55%) (Figure 4b). Thus, *LcSAIN3* overexpression in Arabidopsis reduces sensitivity to salt stress at the seed germination stage.

### 3.5. LcSAIN3 Overexpression in Arabidopsis Enhanced Seed Germination under ABA and Osmotic Stress

**Figure 5:**
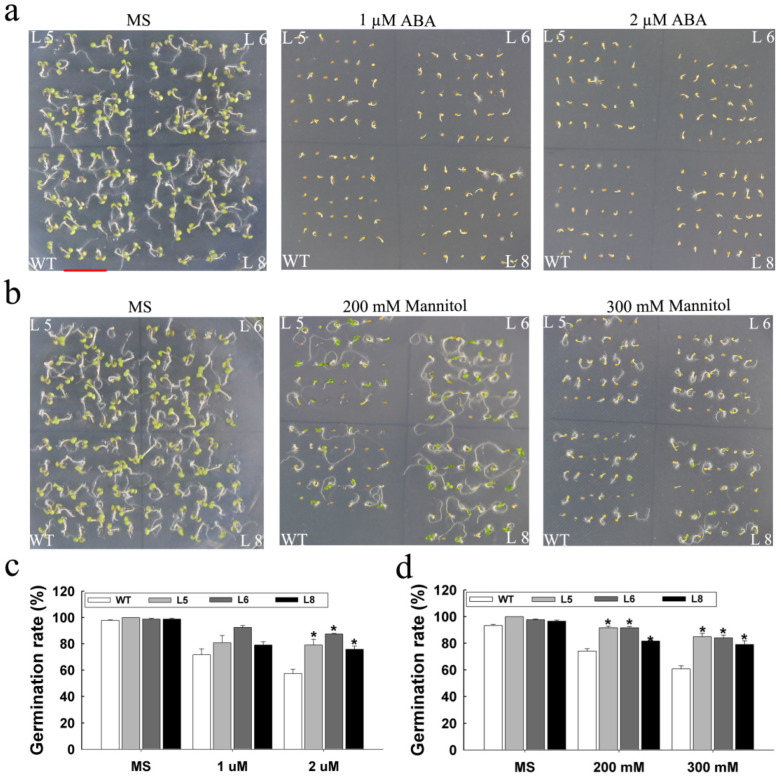
ABA and mannitol stresses tolerance of *LcSAIN3* transgenic Arabidopsis plants at germination stage. (**a**,**c**) seed germination rates of the WT and *LcSAIN3*-expressing lines were measured on MS media and MS media containing 1 and 2 µM ABA. (**b**,**d**) Seed germination rates of the WT and *LcSAIN3*-expressing lines were measured on MS media and MS media containing 200 and 300 mM mannitol. Thirty seeds were allowed to grow for seven days after sowing, and each column represents an average of three replicates; the bars indicate standard deviations (SDs). * indicates significant differences in comparison with the control at *p* < 0.05. Scale bar = 1 cm.

Since the expression of the *LcSAIN3* gene was significantly induced by ABA and osmotic stress (Figure 2), the tolerance to ABA and mannitol of transgenic lines was examined. Likewise, when the Arabidopsis seeds were subjected to 2 μM ABA or 200 and 300 mM mannitol, the germination rate of WT seeds was only 57%, while the germination rates of the three *LcSAIN3*-overexpression lines, i.e., L5, L6, and L8 seeds, were 79%, 87% and 75% under ABA treatment, respectively (*p* < 0.05) (Figure 5a,c). With treatments of 200 and 300 mM mannitol, the germination of WT plants was significantly lower than the transgenic plants (*p* < 0.05) (Figure 5b,d). This implies that over-expression of *LcSAIN3* reduces the sensitivity to ABA and osmotic stresses at the seed germination stage.

### 3.6. LcSAIN3 Overexpression Enhances Tolerance to Salt Stress in Arabidopsis Seedlings

**Figure 6:**
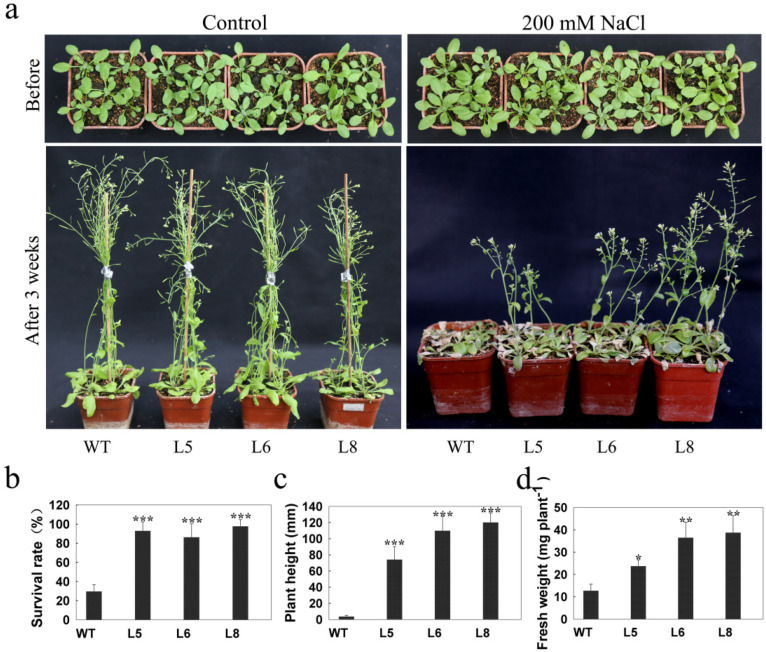
Enhanced tolerances to salt stress in *LcSAIN3*-overexpressing Arabidopsis at the seedling stage. (**a**) Three-week-old seedlings in soil were treated with or without 200 mM NaCl for 3 weeks. The seedling survival rates (**b**) and plant height (**c**) and plant weight (**d**) were scored after the 3-week treatment. The mean and standard error were obtained from three biological replicates, ***, **, and * indicate significant differences at *p* < 0.001, *p* < 0.01, and *p* < 0.05, respectively..

To investigate the performance of *LcSAIN3* transgenic lines in response to salt stress at the seedling stage, 3-week-old Arabidopsis plants were treated with 200 mM NaCl for 3 weeks at 3-day intervals [34]. After exposure to NaCl treatment, the growth of the transgenic lines and WT were all inhibited, but the sensitivity of the *LcSAIN3*-overexpression lines to salt stress was reduced. For example, most of the WT seedlings were bleached and wilted after the 3-week salt treatment; in contrast, most seedlings of three *LcSAIN3*-overexpression lines (L5, L6, and L8) survived and had both green and yellow leaves. The three transgenic lines had significantly higher survival rates (93%, 87%, and 98%) compared to that of WT plants (~30%) (Figure 6a,b). Furthermore, plant height and fresh weight were significantly greater for the transgenic plants than WT plants (Figure 6c,d). These data suggest that overexpression of *LcSAIN3* helped to enhance salt tolerance in transgenic Arabidopsis.

### 3.7. LcSAIN3 Regulates Proline Accumulation and SOD Activity in Response to Salt Stress

**Figure 7:**
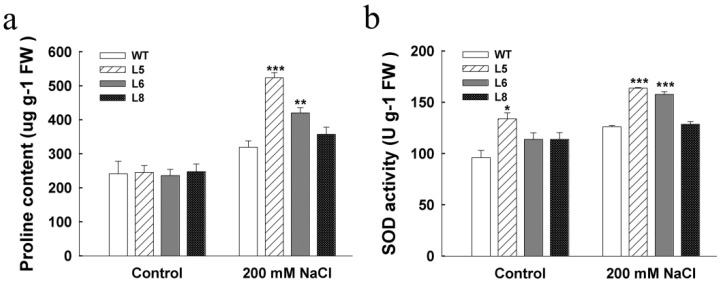
Physiological index analyses of *LcSAIN3*-overexpressing plants. Proline contents (**a**) and antioxidant enzymes levels (**b**) in transgenic and WT seedlings exposed to 200 mM NaCl for 2 d. The bars indicate standard deviations, and the results are from three independent biological replicates. ***, **, and * indicate significant differences in comparison with the control at *p* < 0.001, *p* < 0.01, and *p* < 0.05, respectively.

To further characterize the possible mechanism that may be responsible for improving tolerance of the transgenic plants to salt stress, we assayed proline content and SOD (a major antioxidant enzyme) activity in 3-week-old transgenic and WT seedlings treated with 200 mM NaCl for 2 d. Under the treatment with NaCl, the proline content in both transgenic plants and WT plants increased, but the transgenic plants accumulated a higher level of proline than WT plants, especially in L5 and L6 (Figure 7a). Further, the SOD activity was significantly higher in the transgenic L5 and L6 plants than in the WT plants under salt stress (*p* < 0.01) (Figure 7b). Taken together, our results indicate that *LcSAIN3* overexpression in Arabidopsis could promote proline content and SOD activity under salt stress.

### 3.8. LcSAIN3 Overexpression Alters the Expression of Salt-Responsive Genes in Arabidopsis Plants

**Figure 8:**
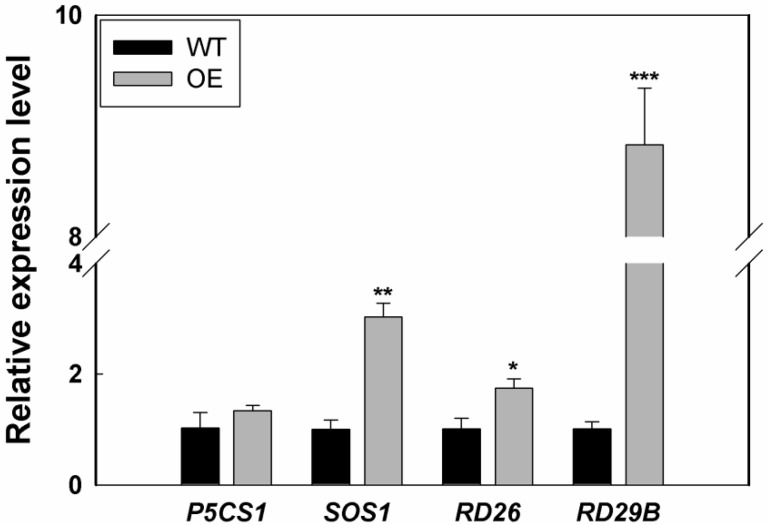
Relative expression levels of five stress-associated genes in the *LcSAIN3*-overexpressing plants compared with wild-type under salt stress. Real-time PCR analysis of six salt-stress-induced genes in transgenic Arabidopsis plants in the presence of 150 mM NaCl. *AtActin2* was used as the reference gene. ***, **, and * indicate significant differences in comparison with the control at *p* < 0.001, *p* < 0.01, and *p* < 0.05, respectively.

To clarify the effect of *LcSAIN3* on the molecular basis of salinity stress response, the expression levels of several known salt-stress-responsive marker genes were compared between the transgenic Arabidopsis (averaged OE lines, average expression levels of transgenic line 5, 6, 8) and WT plants using qRT-PCR under salt stress. As shown in Figure 8, the transcripts of *RD26* and *RD29B* genes increased in the transgenic plants compared with WT plants under salt stress. Similarly, the expression levels of *SOS1* also significantly increased in the transgenic lines (Figure 8). Taken together, these results suggest that *LcSAIN3* may improve the salt stress tolerance of transgenic plants by upregulating the expression of salt-stress-responsive genes.

### 3.9. The Expression of LcSAIN3 in Sheepgrass Germplasms with Different Salt Tolerance

**Figure 9:**
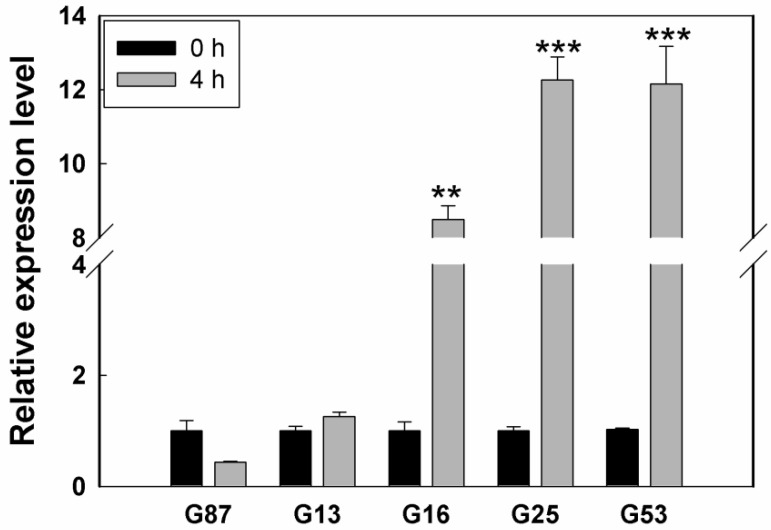
Expression analysis of *LcSAIN3* in sheepgrass germplasms with different salt tolerance. G53, G25 and G16 are salt tolerant germplasms, and G13 and G87 are salt sensitive germplasms. ***, and ** indicate significant differences in comparison with the control at *p* < 0.001, and *p* < 0.01, respectively.

To investigate the expression level of *LcSAIN3* in sheepgrass germplasms with different salt tolerance, we performed qRT-PCR analysis. The results revealed that the relative expression levels of *LcSAIN3* were significantly induced in salt tolerant germplasms (G53, G25, and G16) after 4 h of salt treatment, while the relative expression levels in salt sensitive germplasms (G87, G13) were changed slightly under salt treatment (Figure 9). These results indicate that *LcSAIN3* may play important role in response to salt stress in sheepgrass germplasms. 

## 4. Discussion

Sheepgrass is an important forage grass as well as an environmentally friendly native grass species in China. It has a high yield with high protein content, better palatability, strong regeneration ability, strong cold and drought resistance, as well as salt–alkali resistance [21,23]. Our previous studies have demonstrated that the novel genes *LcFIN1* and *LcFIN2* from sheepgrass enhance tolerance to low temperature in Arabidopsis and rice, while overexpressing *LcSAIN1* and *LcSAIN2* could enhance the salt stress resistance of transgenic plants compared with wild-type plants [24,28,29,30]. The isolated salt-induced gene, *LcSAIN3,* from sheepgrass in the present study has high homology (72%) only with a wheat cDNA clone, WT004_K04 (GenBank ID: AK331493), and the amino acid sequence of LcSAIN3 shows 52% homology with a predicted protein product of *T*. *turgidum* subsp. Durum (Figure 1). In our previous study, two salt-stress-induced LcSAIN1 and LcSAIN2 proteins were mainly localized in the cell nucleus [28,29], while the *LcSAIN3* gene encoded a chloroplast-targeted protein (Figure 3). Further, LcSAIN3 had low homology with LcSAIN1 and LcSAIN2 proteins (Figure S1), and the transcript abundance of the *LcSAIN3* gene was significantly induced by salinity, PEG, and ABA treatments (Figure 2).

The *LcSAIN3* gene is overexpressed in Arabidopsis as genetic transformation in sheepgrass is still very difficult [37]. Our results showed that overexpression of *LcSAIN3* in Arabidopsis led to an increase in tolerance to NaCl, ABA and PEG treatments, as reflected by the increase in the germination rate (Figure 4 and Figure 5). Further, the survival rates, fresh weight, and plant length of transgenic plants under salt stress were markedly increased during the seedling growth stage (Figure 6). Moreover, *LcSAIN3* expression levels were significantly up-regulated in salt tolerant sheepgrass germplasms under salt stress (Figure 9). Thus, our results suggest that *LcSAIN3* may play important role in response to salt stress. Our preliminary results showed that LcSAIN3 is a chloroplast-localized protein (Figure 3). Overexpression of a novel chloroplast protein CEST enhanced the tolerance of transgenic Arabidopsis to multiple environmental stresses [38]; a chloroplast outer envelope protein from *Suaeda salsa* is also involved in oxidative stress tolerance [39]. In addition, overexpression of chloroplast-localized rice OsRH58 also improved seed germination and seedling growth under salt stress conditions by increasing the translation of chloroplast mRNAs [40]. In this study, a novel chloroplast protein, *LcSAIN3*, plays a positive role in the response to salt, ABA, and PEG stresses and could be used as a gene resource to enhance stress tolerance in wheat and other crops. 

The accumulation of proline in plants under salt stress has multiple protective functions, including osmotic protection and ROS scavenging [41]. SOD is proven to be a kind of antioxidant enzyme that plays an important role in scavenging ROS and protects against oxidative stress under salt conditions [20]. Under salt stress conditions, proline levels and SOD activities are higher in the *LcSAIN3*-overexpression lines than in the wild type (Figure 7), suggesting that *LcSAIN3* may be involved in salt stress responses by the accumulation of more proline and higher SOD activity. Other studies have shown that the SOS pathway is a key regulator of Na^+^ homeostasis, for example, via SOS1 [3]. *P5CS1* is a key enzyme in the proline biosynthesis pathway, and it functions as a positive regulator in proline accumulation and plant responses to salt tolerance [13,42]. In the present study, the *AtSOS1* gene is expressed at much higher levels in the *LcSAIN3* transgenic plants than in the WT plants under salt stress and the expression level of *AtP5CS1* was induced lightly (Figure 8). Furthermore, in transgenic Arabidopsis plants overexpressing *LcSAIN3*, the expression of the ABA-dependent genes *AtRD26* and *AtRD29B* are also significantly increased than that in the WT plants under salt stress. These genes have been found to be induced by salinity, and they play important roles in the response to abiotic stress. *AtRD29B* is involved in ABA-dependent signaling pathways [43,44]. These findings indicate that the improved tolerance of the transgenic plants to salinity stress might partly result from the enhanced proline content and SOD activity and the increased expression of salt stress-responsive marker genes (*AtSOS1*, *AtRD26* and *AtRD29B* etc.).

## 5. Conclusions

In summary, we have characterized a novel chloroplast-localized protein, LcSAIN3; the protein plays a positive role in the salt stress pathway. Overexpression of *LcSAIN3* in Arabidopsis elevates seed germination and seedling survival rates under salt stress. The changes of part physiological indices (proline content and SOD activity) and salt-responsive genes (*AtSOS1*, *AtRD29B* etc.) of *LcSAIN3* overexpression plants may give rise to the improving salt tolerance of transgenic plants.

## Data Availability

The gene sequence is available in the NCBI gene database under accession number MN901606 (https://www.ncbi.nlm.nih.gov/nuccore/MN901606, accessed on 3 January 2020).

## Supplementary Materials

The following are available online at https://www.mdpi.com/article/10.3390/genes12121994/s1. All data generated or analyzed during this study are included in this published article and its supplementary information files, namely, Figure S1: Comparison of the amino acid sequences of LcSAIN1, LcSAIN2, and LcSAIN3 proteins and Figure S2: QRT-PCR identification of transgenic Arabidopsis and WT with the *LcSAIN3* gene.

## References

[1] Munns R., Tester M. (2008). Mechanisms of salinity tolerance. Annu. Rev. Plant Biol..

[2] Deinlein U., Stephan A.B., Horie T., Luo W., Xu G., Schroeder J.I. (2014). Plant salt-tolerance mechanisms. Trends Plant Sci..

[3] Isayenkov S.V., Maathuis F.J.M. (2019). Plant Salinity Stress: Many Unanswered Questions Remain. Front Plant Sci..

[4] Xiong L., Schumaker K.S., Zhu J.K. (2002). Cell signaling during cold, drought, and salt stress. Plant Cell.

[5] Reddy A.S., Ali G.S., Celesnik H., Day I.S. (2011). Coping with stresses: Roles of calcium- and calcium/calmodulin-regulated gene expression. Plant Cell.

[6] Zhao C., Zayed O., Zeng F., Liu C., Zhang L., Zhu P., Hsu C.C., Tuncil Y.E., Tao W.A., Carpita N.C. (2019). Arabinose biosynthesis is critical for salt stress tolerance in Arabidopsis. New Phytol..

[7] Ma X., Liang X., Lv S., Guan T., Jiang T., Cheng Y. (2020). Histone deacetylase gene PtHDT902 modifies adventitious root formation and negatively regulates salt stress tolerance in poplar. Plant Sci..

[8] Isayenkov S.V. (2019). Genetic sources for the development of salt tolerance in crops. Plant Growth Regul..

[9] Yang A., Dai X., Zhang W.H. (2012). A R2R3-type MYB gene, OsMYB2, is involved in salt, cold, and dehydration tolerance in rice. J. Exp. Bot..

[10] Hu H., You J., Fang Y., Zhu X., Qi Z., Xiong L. (2008). Characterization of transcription factor gene SNAC2 conferring cold and salt tolerance in rice. Plant Mol. Biol..

[11] Wang C., Lu G., Hao Y., Guo H., Guo Y., Zhao J., Cheng H. (2017). ABP9, a maize bZIP transcription factor, enhances tolerance to salt and drought in transgenic cotton. Planta.

[12] Liang Y., Li X., Zhang D., Gao B., Yang H., Wang Y., Guan K., Wood A.J. (2017). ScDREB8, a novel A-5 type of DREB gene in the desert moss Syntrichia caninervis, confers salt tolerance to Arabidopsis. Plant Physiol. Biochem..

[13] Bo C., Chen H., Luo G., Li W., Zhang X., Ma Q., Cheng B., Cai R. (2020). Maize WRKY114 gene negatively regulates salt-stress tolerance in transgenic rice. Plant Cell Rep..

[14] Hao Y.J., Wei W., Song Q.X., Chen H.W., Zhang Y.Q., Wang F., Zou H.F., Lei G., Tian A.G., Zhang W.K. (2011). Soybean NAC transcription factors promote abiotic stress tolerance and lateral root formation in transgenic plants. Plant J..

[15] He Y., Li W., Lv J., Jia Y., Wang M., Xia G. (2012). Ectopic expression of a wheat MYB transcription factor gene, TaMYB73, improves salinity stress tolerance in Arabidopsis thaliana. J. Exp. Bot..

[16] Peng X.J., Xingyong M., Weihong F., Man S., Liqin C., Alam I., Lee B.H., Dongmei Q., Shihua S., Gongshe L. (2011). Improved drought and salt tolerance of Arabidopsis thaliana by transgenic expression of a novel DREB gene from Leymus chinensis. Plant Cell Rep..

[17] Zhao P., Li X., Jia J., Yuan G., Chen S., Qi D., Cheng L., Liu G. (2019). LcbHLH92 from sheepgrass acts as a negative regulator of anthocyanin/proanthocyandin accumulation and influences seed dormancy. J. Exp. Bot..

[18] Szabados L., Savoure A. (2010). Proline: A multifunctional amino acid. Trends Plant Sci..

[19] Zheng J., Su H., Lin R., Zhang H., Xia K., Jian S., Zhang M. (2019). Isolation and characterization of an atypical LEA gene (IpLEA) from Ipomoea pes-caprae conferring salt/drought and oxidative stress tolerance. Sci. Rep..

[20] Xu Y., Yu Z., Zhang S., Wu C., Yang G., Yan K., Zheng C., Huang J. (2019). CYSTM3 negatively regulates salt stress tolerance in Arabidopsis. Plant Mol. Biol..

[21] Lu P., Magwanga R.O., Kirungu J.N., Hu Y., Dong Q., Cai X., Zhou Z., Wang X., Zhang Z., Hou Y. (2019). Overexpression of Cotton a DTX/MATE Gene Enhances Drought, Salt, and Cold Stress Tolerance in Transgenic Arabidopsis. Front Plant Sci..

[22] Nevo E., Chen G. (2010). Drought and salt tolerances in wild relatives for wheat and barley improvement. Plant Cell Environ..

[23] Chen S., Huang X., Yan X., Liang Y., Wang Y., Li X., Peng X., Ma X., Zhang L., Cai Y. (2013). Transcriptome analysis in sheepgrass (Leymus chinensis): A dominant perennial grass of the Eurasian Steppe. PLoS ONE.

[24] Gao Q., Li X., Jia J., Zhao P., Liu P., Liu Z., Ge L., Chen S., Qi D., Deng B. (2016). Overexpression of a novel cold-responsive transcript factor LcFIN1 from sheepgrass enhances tolerance to low temperature stress in transgenic plants. Plant Biotechnol. J..

[25] Cheng L., Li X., Huang X., Ma T., Liang Y., Ma X., Peng X., Jia J., Chen S., Chen Y. (2013). Overexpression of sheepgrass R1-MYB transcription factor LcMYB1 confers salt tolerance in transgenic Arabidopsis. Plant Physiol. Biochem..

[26] Ma T., Li M., Zhao A., Xu X., Liu G., Cheng L. (2014). LcWRKY5: An unknown function gene from sheepgrass improves drought tolerance in transgenic Arabidopsis. Plant Cell Rep..

[27] Liu Z., Yuan G., Liu S., Jia J., Cheng L., Qi D., Shen S., Peng X., Liu G. (2017). Identified of a novel cis-element regulating the alternative splicing of LcDREB2. Sci. Rep..

[28] Li X., Hou S., Gao Q., Zhao P., Chen S., Qi D., Lee B.H., Cheng L., Liu G. (2013). LcSAIN1, a novel salt-induced gene from sheepgrass, confers salt stress tolerance in transgenic Arabidopsis and rice. Plant Cell Physiol..

[29] Li X., Gao Q., Liang Y., Ma T., Cheng L., Qi D., Liu H., Xu X., Chen S., Liu G. (2013). A novel salt-induced gene from sheepgrass, LcSAIN2, enhances salt tolerance in transgenic Arabidopsis. Plant Physiol. Biochem..

[30] Li X., Yang W., Liu S., Li X.Q., Jia J., Zhao P., Cheng L., Qi D., Chen S., Liu G. (2019). LcFIN2, a novel chloroplast protein gene from sheepgrass, enhances tolerance to low temperature in Arabidopsis and rice. Physiol. Plant..

[31] Chou K.C., Shen H.B. (2010). Plant-mPLoc: A top-down strategy to augment the power for predicting plant protein subcellular localization. PLoS ONE.

[32] Livak K.J., Schmittgen T.D. (2001). Analysis of relative gene expression data using real-time quantitative PCR and the 2(T)(-Delta Delta C) method. Methods.

[33] Clough S.J., Bent A.F. (1998). Floral dip: A simplified method for Agrobacterium-mediated transformation of Arabidopsis thaliana. Plant J..

[34] Zhao Y., Yang Z., Ding Y., Liu L., Han X., Zhan J., Wei X., Diao Y., Qin W., Wang P. (2019). Over-expression of an R2R3 MYB Gene, GhMYB73, increases tolerance to salt stress in transgenic Arabidopsis. Plant Sci..

[35] Shan D.P., Huang J.G., Yang Y.T., Guo Y.H., Wu C.A., Yang G.D., Gao Z., Zheng C.C. (2007). Cotton GhDREB1 increases plant tolerance to low temperature and is negatively regulated by gibberellic acid. New Phytol..

[36] Durak I., Yurtarslanl Z., Canbolat O., Akyol O. (1993). A methodological approach to superoxide dismutase (SOD) activity assay based on inhibition of nitroblue tetrazolium (NBT) reduction. Clin. Chim. Acta.

[37] Wang L., Li X., Chen S., Liu G. (2009). Enhanced drought tolerance in transgenic Leymus chinensis plants with constitutively expressed wheat TaLEA3. Biotechnol. Lett..

[38] Yokotani N., Higuchi M., Kondou Y., Ichikawa T., Iwabuchi M., Hirochika H., Matsui M., Oda K. (2011). A novel chloroplast protein, CEST induces tolerance to multiple environmental stresses and reduces photooxidative damage in transgenic Arabidopsis. J. Exp. Bot..

[39] Wang F., Yang C.L., Wang L.L., Zhong N.Q., Wu X.M., Han L.B., Xia G.X. (2012). Heterologous expression of a chloroplast outer envelope protein from Suaeda salsa confers oxidative stress tolerance and induces chloroplast aggregation in transgenic Arabidopsis plants. Plant Cell Environ..

[40] Nawaz G., Kang H. (2019). Rice OsRH58, a chloroplast DEAD-box RNA helicase, improves salt or drought stress tolerance in Arabidopsis by affecting chloroplast translation. BMC Plant Biol..

[41] Zsigmond L., Szepesi A., Tari I., Rigo G., Kiraly A., Szabados L. (2012). Overexpression of the mitochondrial PPR40 gene improves salt tolerance in Arabidopsis. Plant Sci..

[42] Xu N., Chu Y., Chen H., Li X., Wu Q., Jin L., Wang G., Huang J. (2018). Rice transcription factor OsMADS25 modulates root growth and confers salinity tolerance via the ABA-mediated regulatory pathway and ROS scavenging. PLoS Genet..

[43] Msanne J., Lin J., Stone J.M., Awada T. (2011). Characterization of abiotic stress-responsive Arabidopsis thaliana RD29A and RD29B genes and evaluation of transgenes. Planta.

[44] Han G., Yuan F., Guo J., Zhang Y., Sui N., Wang B. (2019). AtSIZ1 improves salt tolerance by maintaining ionic homeostasis and osmotic balance in Arabidopsis. Plant Sci..

