# Supplementary material for: Ectopic Expression of a Salt-Inducible Gene, LcSAIN3, from Sheepgrass Improves Seed Germination and Seedling Growth under Salt Stress in Arabidopsis
Source: Genes (Basel). 2021 Dec 16;12(12):1994. doi: 10.3390/genes12121994 (PMC8701343; doi:10.3390/genes12121994)

Figure S1

```
LcSAIN1    ...............................MICAVAEVAGALVVGESGMDGSVNCLVP.     28
LcSAIN2    ....................MGDVSTIQRPPGTAAKQGDQVGELVSAGWTNDSHDMYISS     40
LcSAIN3    MARCAAMATGVMPLMCCLPLLPCSKRWPPVWSFCPPPWWRRWEDDGSDVSSSNKHAGVPD     60

LcSAIN1    .VLQVRPHLLLCFDG.........LRG..............DGDGVEKMWVSSRRRLVSS     64
LcSAIN2    MEASFMQRLRGQHHHAAADRNMTHVG..............AGHGLKSHQQGASDNVSRS     86
LcSAIN3    RDTQVISFLSSCCHGGGEGGGGRSLRGFVFWCATPSDERFWRGGSLPRIWWRTSSSLTLV    120

LcSAIN1    SFSLVLDD.ERWRLLKLHPSHGCDVS.............GRGPASSISPVTVLVEGRPLS    110
LcSAIN2    RDVGARGLPEDPWARRFKPRDSVMNRR............GDGVGASDG.....ESGTDTV    129
LcSAIN3    AVTVVVEL.LRWIHWKLDDADGVRQHRRRWIWRSAGAHVGRWPASSDEPLYPLAEWRHSS    179

LcSAIN1    FLPASVPK.GRQFQPQLGGRSLQVLQLWRPRRTKWSVPGAGAIQSEEMLLGTRSRFLVLV    169
LcSAIN2    HVTAPTPKHGRGVNSCVG....GNLVEKTSEVSGQHFPEDEVHSAAQVSKSCKKRRPASS    185
LcSAIN3    FLPASMPK.GRQYCFWIR...VHGMVSWRSRRTKWPCLRR...RRDWFHSGTVSDLIAIL    232

LcSAIN1    RGPFCISQGLCCSFQFLLGFLVKCTAPTF                                  198
LcSAIN2    ........TAAGSFISMFGDKRW......                                  200
LcSAIN3    ..........VCNFLFVLGPDVICVFMSI                                  251
```

Figure S2

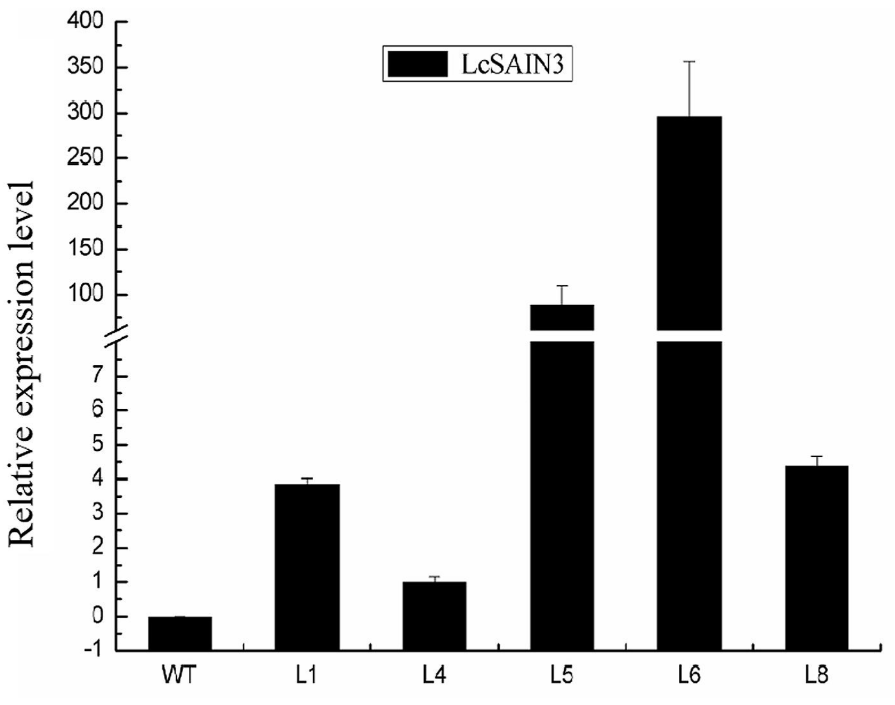

Supplement: Supplementary file 1 [file genes-12-01994-s001.zip › genes-1460833-supplementary.pdf]
